# Apple Pomace as Valuable Food Ingredient for Enhancing Nutritional and Antioxidant Properties of Italian Salami

**DOI:** 10.3390/antiox11071221

**Published:** 2022-06-22

**Authors:** Luca Grispoldi, Federica Ianni, Francesca Blasi, Luna Pollini, Silvia Crotti, Deborah Cruciani, Beniamino Terzo Cenci-Goga, Lina Cossignani

**Affiliations:** 1 Department of Veterinary Medicine, University of Perugia, 06126 Perugia, Italy; luca.grispoldi@unipg.it; 2 Department of Pharmaceutical Sciences, University of Perugia, 06126 Perugia, Italy; federica.ianni@unipg.it (F.I.); luna.pollini@studenti.unipg.it (L.P.); lina.cossignani@unipg.it (L.C.); 3 Istituto Zooprofilattico Sperimentale dell’Umbria e delle Marche “Togo Rosati”, 06126 Perugia, Italy; s.crotti@izsum.it (S.C.); d.cruciani@izsum.it (D.C.); 4 Center for Perinatal and Reproductive Medicine, University of Perugia, Santa Maria della Misericordia University Hospital, 06132 Perugia, Italy

**Keywords:** apple pomace, waste, phenols, antioxidant activity, salami, fortified meat, sensory evaluation

## Abstract

Nowadays, food fortification with bioactive compounds deriving from agri-food waste is of great interest all over the world. In this work, apple pomace (AP), the most abundant by-product of apple juice manufacturing, was characterised by chemical, chromatographic and spectrophotometric analyses. AP showed valuable antioxidant activity, due to the presence of phenolic compounds (8.56 mg gallic acid equivalents/g), including quercetin-3-*O*-galactoside, quercetin-3-*O*-arabinofuranoside, and phloridzin. Dried AP, at 7% and 14%, was added to pork meat to produce Italian salami, then subjected to 25 days of ripening. Physicochemical, colorimetric and microbiological analyses were carried out at days 0, 5, 11, 19 and 25, while nutritional and sensory evaluations were performed at the end of the ripening. The overall acceptability was slightly higher for 7% AP compared to 14% AP sample, and generally the replacement of a percentage of meat with apple pomace allowed the production of salami with sensory properties comparable to those obtained with classic recipes. The improved fibre and phenol content, together with the lower fat and calories, represent the most interesting characteristics of fortified salami. The results confirm that the addition of AP represents a valid approach to adding healthy compounds to salami.

## 1. Introduction

In recent years, consumers have become increasingly aware that proper nutrition is directly linked to their wellbeing and can prevent diet-related diseases. Subsequently, consumer demand for healthier foods has increased, especially after the COVID-19 pandemic [1]. Moreover, another important worldwide problem is the generation and management of food waste; therefore, the food industry has been trying to address this big challenge and reduce secondary pollution by reusing food residues [2].

Residues from the agri-food industry, i.e., by-products and waste, are valuable sources for the recovery of bioactive compounds, among which polyphenols and fibre are applicable in the food industry as natural functional food additives and ingredients. In fact, recent studies have reported that these compounds possess health-promoting effects, including antioxidant, anti-inflammatory, and immunomodulatory properties [3].

As an example, apple pomace (AP), the primary by-product of apple juice manufacturing commonly used as animal feed and biofuel, was proposed as a source of dietary fibre and phenols [4]. Many research studies focused on the extraction and characterisation of bioactive compounds from apple pomace [5,6,7,8]. For example, Candrawinata et al. [5] optimised by response surface methodology the aqueous extraction of phenolic compounds from AP, evaluating the effects of temperature, time, and the pomace/water ratio. Persic and coworkers analysed apple fruit, juice and pomace and the correlation between phenolic content, enzymatic activity, and browning [6]. More recently, the kinetic modelling of the solid–liquid extraction of total phenolic content from AP was assessed by Hobbi et al. [7], while AP was used as a source of bioactive phenols to enrich gluten-free breads [8].

Nowadays, food functionalisation is a growing market for which the food industry requires new bioactive ingredients useful for the development of innovative functional products, both of animal and plant origin. In this regard, much attention has been recently paid to natural compounds deriving from vegetable waste and their association with high functionality and/or bioactivity [2].

Numerous steps forward in the valorisation of by-products and waste from the agri-food processing industry have been made, and among these, their use for the fortification of animal-derived products [9]. In fact, despite growing evidence supporting an association between processed meat intake and chronic diseases, this type of food continues to be consumed in high quantities all around the world. In Europe, the processed meat market segment is expected to grow annually by 0.78% (compounded average growth rate, CAGR, 2022–2027), with an average volume per person expected to amount to 19.2 kg in 2022, based on OECD–FAO agriculture statistics [10].

The magnitude of processed meat consumption offers the opportunity to develop functional foods by adding fibre and antioxidants that have positive effects on health, but are not consumed in sufficient quantities in most countries. In fact, generally, low consumption of vegetables and fruits implies an intake of fibre and antioxidants lower than the levels recommended by the WHO, although these functional components are protective against cardiovascular disease, metabolic syndrome and chronic diseases [11]. Furthermore, the replacement of chemical additives with natural compounds in meat preparations or the addition of natural extracts or preparations are a matter of great interest today, both for consumers and for specialists in the food sector. In fact, this approach allows not only an increase in the intake of dietary fibre and antioxidants and reduction of fats, but also offers to improve the shelf-life and rheological properties of the supplemented products [12].

For example, Balzan et al. [13] studied the effect of an extract rich in phenols obtained from olive vegetation water, an agricultural by-product, on raw and cooked fresh pork salami prepared without chemical additives. Kharrat et al. [14] studied the substitution of some synthetic additives by a natural extract from red prickly pear (2.5%) rich in bioactive polysaccharides and phenols that displayed strong antioxidant and antimicrobial activities in salami. Peanut skin extract at 0.1 ppm had a protective effect on salami, evaluated by chemical indicators and descriptive sensory attributes, prolonging its shelf life during 42 days of storage [15]. Recently, the incorporation of green tea leaf powder effectively inhibited bacterial growth and lipid oxidation in lamb salami, even if the sensory quality was not improved [16].

Buffalo meat salami supplemented with 6% AP powder showed improved physicochemical and sensory properties [17]. Choi and coworkers studied the effect of AP fibre and pork fat levels on quality characteristics of uncured, reduced-fat chicken salami [18]. Similarly, Rather et al. [19] used AP powder as a fat replacer in goshtaba, a traditional Indian meat product. In a previous work, Pollini et al. [20] used AP for beef burger fortification and found that the functionalised products had improved fibre and phenol content and flavour.

In this paper, after an initial chemical and nutritional characterisation of dried AP by spectrophotometry and high-performance liquid chromatography (HPLC) analyses, two different percentages of this by-product (7% and 14%) were added to meat batches in salami production in order to produce fortified products, with improved their nutritional and functional characteristics. The fortified salami products were analysed for their chemical composition, physical and functional properties, colour, microbiological characteristics, and sensory and textural properties and compared with nonfortified salami (0%, control).

## 2. Materials and Methods

### 2.1. Materials, Reagents, Samples

Folin and Ciocalteu’s phenol reagent, 2,2′-azino-bis(3-ethylbenzothiazoline-6-sulphonic acid) diammonium salt (ABTS), 2,2-diphenyl-1-picrylhydrazyl (DPPH radical), gallic acid, (±)-6-hydroxy-2,5,7,8-tetramethylchromane-2-carboxylic acid (Trolox), methanol, and ethanol were from Sigma-Aldrich (Milan, Italy). Edible-grade ascorbic acid, analysed according to Ph.Eur.9.3, was from Caelo Caesar and Loretz, GMBH (Hilden, Germany). Megazyme^®^ kit K-TDFR-100A/K-TDFR-200A 08/16 was purchased from Megazyme (Megazyme International Ireland, Bray, Ireland). Red Delicious apples were purchased in a local supermarket.

### 2.2. Preparation of AP

Apples were washed with water and cut into pieces, and separated from the seeds and petioles. The pieces were dipped in edible-grade 1% ascorbic acid solution before juice extraction. AP was obtained with a domestic fruit juice extractor (R.G.V., Como, Italy) and then dried at 55 °C until constant weight. Finally, it was milled with a blender (Oster, model n. 869-50R, Milwaukee, WI, USA) to obtain a homogeneous dried powder. A part of dried AP was stored in amber glass jar at room temperature in the dark, until subsequent analytical characterisation as reported in the following Section 2.3. A portion of the dried AP was rehydrated with distilled water prior to being added to meat for salami preparation. The production of salami is reported in Section 2.4.

### 2.3. Chemical and Nutritional Characterisation of AP

The proximate composition of AP was determined according to Association of Official Analytical Chemists (AOAC) procedures [21]. Protein was determined as total nitrogen content (N × 6.25) using the Kjeldahl procedure (method n. 920.87). Moisture and ash contents were determined according to methods n. 925.10 and 923.03, respectively. Total dietary fibre was determined according to AOAC method n. 991.43, using Megazyme^®^ enzymatic gravity kit.

The bioactives in AP were extracted by ultrasound assisted extraction (UAE) following the conditions used in a previous paper (ethanol:water, 50:50 *v/v*; solid/liquid ratio, 1:10 *w/v*; 45 °C for 45 min; ultrasonic bath, AU-65 model, ultrasonic/heating power 180 W, ArgoLab, Carpi, Italy) [20]. AP extract was then analysed to evaluate the total phenol content (TPC) and in vitro antioxidant activity [20]. The determination of TPC was performed according to Pagano et al. [22] with slight modifications. Folin and Ciocalteu’s phenol reagent was used, and the absorbance was measured at 765 nm. The TPC was reported as mg gallic acid equivalents per gram of dry weight AP (mg GAE/g DW). The free radical-scavenging activity was measured using ABTS assay according to a previous paper [23] with slight modifications. A freshly prepared ABTS solution was used, and the absorbance was measured at 734 nm. The free radical-scavenging activity of the sample was also evaluated by using the DPPH reagent, and the absorbance was measured at 517 nm [23]. Finally, the reducing capacity of the extracts was evaluated using the ferric reducing antioxidant power (FRAP) assay according to Pollini et al. [20], with slight modifications. The FRAP reagent was used, and the absorbance was measured at 593 nm. Results of ABTS, DPPH, and FRAP assays were reported as mg of Trolox equivalents per gram of dry weight AP (mg TE/g DW).

The qualitative and quantitative profiles of phenolic compounds were obtained by HPLC-UV analysis, as reported in a previous work [20].

### 2.4. Production of Italian Salami Fortified with AP

Salami was prepared at the pilot plant of the Laboratorio di Ispezione degli Alimenti di Origine Animale (Department of Veterinary Medicine, University of Perugia, Perugia, Italy), according to a procedure handed down among butchers for centuries. For each replication, the meat used came from the same farm, and all of the animals were “suino pesante italiano tipico” with a live weight of over 150 kg and an age of over 9 months. Meat, shoulder and flank (90%) and hind fat (10%) were minced and blended with the ingredients (sodium chloride 30 g/kg, pepper 5 g/kg, ascorbic acid 2 g/kg).

The mixture was stuffed under vacuum into a natural, 30 mm-diameter swine casing, and the salami (10 cm in length) was suspended in a conditioned chamber for fermentation and ripening. The temperature and relative humidity were recorded by the chamber data-logger during the entire period (Figure S1).

**Figure 1:**
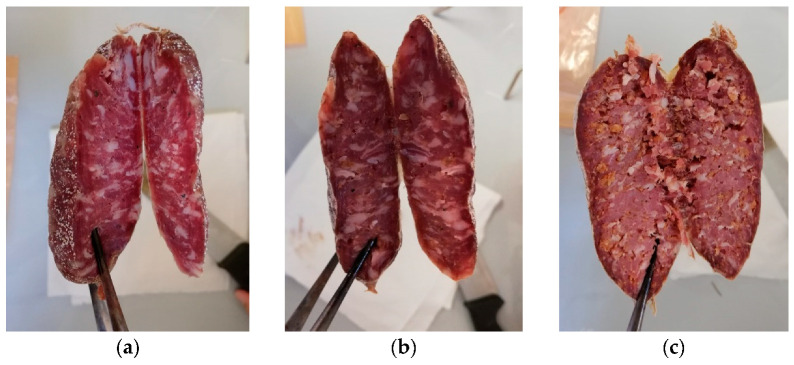
Pictures of the samples at 11-day ripening. (**a**) C, control (no added AP, 0%); (**b**) 7% AP, salami with 7% added AP; (**c**) 14% AP, salami with 14% added AP.

The study was performed in three different replications on three different days. Three batches were produced for each replication: C, control (0% AP); 7% AP, salami with 7% added AP; 14%AP, salami with 14% added AP (Figure 1).

Sampling (three salami samples per group) was performed at the beginning of the ripening (T0) and then after 5, 11, 19 and 25 days (T5, T11, T19, T25).

The proximate composition of the starting meat mixture was characterised according to AOAC methods (n. 981.10 for moisture, n. 991.36 for fat, n. 950.46 for ash, and n. 920.153/923.03 for protein) [21]. The carbohydrate fraction was determined by difference.

The nutritional composition of salami at the end of the ripening (T25) was obtained using the above-cited AOAC methods, while TPC and antioxidant activity were evaluated using the procedures reported in Section 2.3 after UAE extraction with methanol:water, 70:30 *v/v*, for 30 min at room temperature.

### 2.5. Physicochemical Analysis of Fortified Salami

To measure the pH, an S40 Seven-Multi digital pH-meter (Mettler-Toledo Italia, Novate Milanese, Italy) was used after mixing 5 g of salami with 50 mL of distilled water and then centrifuging (Neya A 8-50) at 2150× *g* for 5 min. A dew-point hygrometer HygroLab 3 (Rotronic, Huntington, NY, USA) was used to measure water activity (a_w_).

An instrumental measurement of the samples’ hardness was also carried out at the end of ripening by using a digital dynamometer FL 100 (Sauter Italia, Cinisello Balsamo, Milan, Italy). A modified version of the Warner–Bratzler shear force method was used: all samples were cut into cylinders of 2 cm in height and 1 cm in diameter, and then compressed with a wedge-shaped probe.

### 2.6. Colour Analysis of Fortified Salami

The samples’ colour was measured using the ColorMeter RGB Colorimeter app (White Marten GmbH, Stuttgart, Germany) for iOS on an iPhone XS with iOS 13.7. The app was calibrated against a reference colorimeter, a Chroma Meter Minolta CR 200 (Konica Minolta Inc., Tokyo, Japan). This way we were able to measure the average colour of the entire product in order to replicate the consumer’s perception. The methodology used has already been described by the authors in previous manuscripts [20,24]. The CIELAB scale was used to express results. The CIELAB colour space covers the entire range of human colour perception and it is based on the opponent colour model of human vision. It expresses colour as three values: L* for lightness, a* for red-green opponent colours and b* for yellow-blue opponent colours.

### 2.7. Microbiological Analysis of Fortified Salami

For each analysis, eight bacterial populations were evaluated: total aerobic mesophilic flora, *Lactobacillus* spp., *Lactococcus* spp., enterococci, *Staphylococcus* spp., *Enterobacteriaceae*, total coliforms and *Pseudomonas* spp. The methodology used for the microbiological analysis has already been described by the authors in a previous work [25]. At the end, the number of colonies was converted to the log of colony forming units per gram (log CFU/g), and the mean was calculated for each analysis. Sensitivity for spread plate was 10^2^ CFU/g and for pour plate was 10 CFU/g, and the 95% confidence limit was set between ±37% and ±12% (i.e., plates with a number of CFU ranging from 30 to 300). Therefore, all plates with less than 30 CFU were not used for data analysis, and when this applied to the lowest dilution, results were recorded as <300 for pour plate and <3000 for spread plate [26]. Fungal colonies with visibly different morphological appearance were isolated from the exterior of the salami and identified by PCR and genome sequencing [27].

### 2.8. Sensory Evaluation of Fortified Salami

A panel test was performed. The panel consisted of 16 previously trained assessors. The tasters were asked to test salami for the following characteristics: colour uniformity, colour intensity, fat/lean connection, fat/lean distribution, odour (global intensity), mouldy odour, acid flavour, rancid flavour, bitter flavour, salty flavour, mouldy flavour, spicy flavour, flavour intensity, elasticity, hardness, cohesiveness, chewiness, juiciness, fattiness and overall acceptability. Each assessor was given sheets with a 7-point (unnumbered to avoid biased assessment) scale for each characteristic: 7 = maximum intensity and 1 = minimum intensity. The evaluations were performed in individual booths, built according to the criteria of the International Standard Organization [28]. Water and unsalted bread were provided to cleanse the palate between samples. Assessments were carried out under natural light at a room temperature of 20 ± 2 °C. The individual scores by each assessor were then averaged to give a score for the taste panel as a whole. Three evaluations for each different batch of salami were performed. Each evaluation was carried out in different test sessions at the same time of day, between 10 and 12 a.m.

### 2.9. Statistical Analysis

All analytical determinations were performed at least three times, and the results were reported as mean value and standard deviation (SD). Statistical analysis was performed with GraphPad Prism, version 6.0 h, for Mac OS X (GraphPad, San Diego, CA, USA). Two-way analysis of variance (ANOVA) followed by the Tukey’s multiple comparisons test was performed considering the product type as the treatment. A *p*-value < 0.05 was considered to be significant.

## 3. Results and Discussion

### 3.1. Proximate Composition and Chemical Characterisation of AP

AP, the main by-product obtained after crushing and pressing apples for juice production, is an abundant residue, rich in nutrients and bioactives. Initially, dried AP was analysed for its proximate composition, which showed average contents of 5.90% ± 0.05, 2.73% ± 0.04, and 1.29% ± 0.42 for moisture, protein, and ash, respectively. The total amount of fibre, the major component of AP, was about 40.68%, a value determined by an enzymatic procedure kit as the sum of insoluble (32.81% ± 0.27) and soluble (7.87% ± 0.43) fractions. Wide variability was found for AP composition, mainly related to apple cultivar, harvesting and processing, as recently reviewed by Antonic et al. [4].

As regards the characterisation of bioactives, in a recent paper [20] it was concluded that UAE was a successful technique for extracting phenol compounds. For this reason, in this paper the same extraction conditions were applied, and the extract was subjected to spectrophotometric assays for TPC and antioxidant activity evaluation, and to HPLC-UV analysis for phenol qualitative and quantitative characterisation.

Table 1 shows the values of TPC and the results of antioxidant assays (ABTS, DPPH and FRAP) of UAE extract. The AP extract showed a TPC value of 8.56 mg GAE/g DW, comparable to the results obtained in previous works [20,29,30,31]. Finely ground AP incorporated in wheat flour showed a TPC value of 10.16 mg GAE/g [29]. AP samples obtained from industrial pomace showed a wide range of TPC values (3.9–13.9 g GAE/kg) due to different cultivars and technological processing [30]. Ferrentino et al. [31] used supercritical fluid for extracting phenols from AP and found a TPC value of 6.41 mg GAE/g for freeze-dried AP, a value that increased to 8.87 mg GAE/g using 5% ethanol as co-solvent. Antonic et al. [4] reported in a recent review a quantity of phenolics in dried AP ranging from 0.17 to 0.99 g/100 g.

Then, the AP extract was subjected to spectrophotometric analyses in order to evaluate its antioxidant activity, by using three complementary in vitro assays: the ABTS and DPPH assays measure the ability of antioxidants to scavenge chromogen ABTS and DPPH free radical, respectively, while the FRAP assay determines the reducing capacity of the extract. The results for the antiradical and reducing activity of AP extract are shown in Table 1.

Similar values for ABTS, DPPH and FRAP were obtained in a previous work [20] that reported antioxidant properties of AP obtained from apples of the same cultivar (Red Delicious). Generally, considering the different units of measurement for the antioxidant assay results, there are objective difficulties in making comparisons between the results of different investigations. For example, micronised AP used as emulsifier for food Pickering emulsion showed FRAP values from 52.04 to 59.56 mmol Fe^2+^ equivalents/100 g, ABTS values from 33.84 to 38.19 mg TE/g, and DPPH values from 2.33 to 3.80 mg TE/g, on the basis of different particle sizes of samples [32]. Ferrentino et al. [31] reported a DPPH value of 3.24 mg TE/g for freeze-dried AP, a value that increased up to 5.99 mg TE/g using 5% ethanol as co-solvent.

Furthermore, an HPLC-UV analysis of the extract was carried out to determine the phenolic compounds responsible for the antioxidant activity. The procedure, validated in a previous paper [20], allowed us to quantify quercetin derivatives (galactoside, arabinofuranoside, rhamnoside, xyloside, glucoside, pentoside), phloridzin and chlorogenic acid. Table 2 shows their contents, and it can be observed that the main compound was quercetin-3-*O*-galactoside (1039.54 mg/kg DW), followed by quercetin-3-*O*-arabinofuranoside (563.69 mg/kg DW) and phloridzin (396.18 mg/kg DW), while the main phenolic acid was chlorogenic acid (37.17 mg/kg DW). In Figure S2, the HPLC-UV profile of the AP extract with the identified compounds is shown. Quercetin-3-*O*-rutinoside (rutin) and quercetin-3-O-arabinopiranoside were below the limit of quantification.

The qualitative and quantitative composition of phenolic compounds in AP varied greatly, as reported by Antonic et al. [4]. These authors reported a wide range of contents for phloridzin (8–1435.4 mg/kg DW), chlorogenic acid (26–2298 mg/kg DW), and quercitrin (69–373.8 mg/kg DW), together with other compounds (catechin, epicatechin, caffeic acid, procyanidin B2, and hyperin). García et al. [30] also reported many bioactive compounds belonging to phenolic acid, flavanol, dihydrochalcone, and flavonol classes with a wide range of contents. For example, chlorogenic acid values ranged from 393.2 up to 1415.5 mg/kg, while phloridzin ranged from 587.2 to 1435.4 mg/kg. Ćetković et al. [33] studied five varieties of apples and found rutin ranging from 0.211 to 0.477 mg/g, and chlorogenic acid ranging from 0.030 to 0.176 mg/g.

### 3.2. Physicochemical and Colour Analysis of Fortified Salami

Initially the proximate composition of the starting meat mixture was determined, and the results were: moisture (66.19 ± 1.57 g/100 g meat), fat (11.21 ± 0.54 g/100 g meat), ash (3.18 ± 0.05 g 100/g meat), protein (19.04 ± 0.72 g/100 g meat), carbohydrates (1.88 ± 0.24 g/100 g meat). Physicochemical and colour analyses of rehydrated apple pomace were also performed, and the results were: L* coordinate 17.67 ± 0.58, a* coordinate 11.00 ± 0.00, b* coordinate 10.67 ± 0.58, a_w_ 0.96 ± 0.00 and pH 3.22 ± 0.01.

Then, physicochemical and colour analyses of fortified salami were carried out. The pH values at the beginning of the experiment (T0) ranged between 6.48 ± 0.01 (control) and 5.95 ± 0.02 (salami with 14% AP). Generally, the pH values decreased after 5 days, and then increased up to the end of the ripening. At this time, the pH values were 6.88, 6.27, and 6.36 for C, 7% AP, and 14% AP, respectively (Table 3). The pH value after 5 and 11 days was lower with respect to time 0, probably due to mild sourness of AP, then the pH value increased probably due to the ripening process. A decrease in pH after AP incorporation into meat was also reported by other authors [17,18,19,34]. Younis and Ahmad [17] reported a pH value of 6.17 for control, and 6.03 for buffalo sausage with 6% added AP powder. Rather et al. [19] found a pH value of 5.67 for control and 5.53 for uncooked goshtaba containing 5% AP powder, or 5.61 vs. 5.34 when the same products were cooked. In other investigations, Choi and collaborators found higher pH values in control batches when compared with batches (meat batter, frankfurter) produced with AP fibre [18] and frankfurters produced with dietary fibre extracted from makgeolli lees [34].

Water activity (a_w_) had similar values from day 0 to day 5 in all samples. This could be related to the high humidity programmed in the ripening chamber at this step. After day 5, the a_w_ of samples decreased, reaching values of 0.79, 0.77 and 0.75 for control salami, salami with 7% AP, and salami with 14% AP, respectively, at the end of ripening.

The instrumental measurement of hardness (Figure S3) showed the following results: 61.98 ± 10.25 N for control salami, 52.93 ± 2.07 N for salami with 7% AP, and 80.07 ± 21.74 N for salami with 14% AP. These results are in line with the sensory evaluation where assessors assigned higher values for hardness to 14% AP salami, while control and 7% AP salami obtained lower and similar scores. Results for the evaluation of shear force in Newtons obtained in our study were similar to those reported by other authors for similar products, such as chorizo de Pamplona [35].

The results of the colorimetric analysis are shown in Table 3. Among the physical characteristics, colour has considerable importance in the choices made by consumers. The CIELAB scale provides colorimetric attributes through three parameters: L* (lightness), a* (redness) and b* (yellowness). In meat products, the a* coordinate has particular importance, as it is well-known that consumers tend to prefer products with higher redness, which is commonly considered as an index of the quality and healthiness of the product itself. In our study, the a* coordinate was on average higher in the control than in the groups with the addition of AP.

This difference was statistically significant (*p* < 0.05) at all analysis time points except T19. A similar behaviour was observed in the b* coordinate, but in this case, the increase in the percentage of AP led to an average increase in yellowness, particularly evident in samples containing 14% AP. These results show how the effect on colour was more evident as the amount of AP increased. Similar results have been reported in the literature by other authors, such as Syuhairah et al. [36]. These authors report how the replacement of different percentages of meat with products of plant origin (spinach, purple cabbage, carrot, capsicum and oyster mushroom) involved colour changes in the final product, in particular, a decrease in a* coordinate values and an increase in b* coordinate values. This effect is certainly linked to the original colour of the vegetable product used, which in our case tended to yellow. Choi et al. [18] published a study related to the production of chicken meat-based sausages with the addition of AP. They reported that lightness and yellowness values exhibited a tendency to increase with increasing AP content, while redness values decreased. Very similar results were reported by Park et al. [37] and Choi et al. [34] in pork products.

### 3.3. Nutritional Composition and Antioxidant Properties of Salami

**Figure 2:**
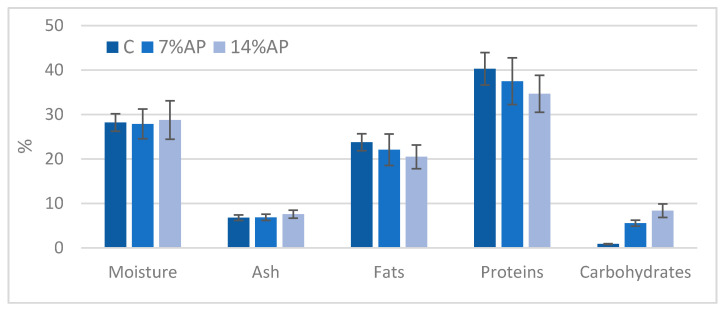
Proximate composition (g/100 g) of fortified salami at the end of ripening (mean values ± SD, n = 3). C, control (no added AP, 0%); 7% AP, salami with 7% added AP; 14% AP, salami with 14% added AP.

The proximate composition of salami at the end of ripening is given in Figure 2. The moisture and ash content in the C and 7% AP samples was similar and slightly lower than in the 14% AP sample. In contrast, fat and protein content showed a decrease as the percentage of AP increased (fat: 23.8 vs. 20.5; proteins: 40.3 vs. 34.7). The carbohydrate content (simple and complex) increased from 0.9 in the C sample to 8.4 in the 14% AP sample. This increase was due to the addition of AP containing complex carbohydrates, and in particular, an abundant amount of fibre. Obviously, the caloric energy (kcal/100 g) also decreased, from 379.00 kcal/100 g in control to 371.30 in the 7% AP sample, down to 356.90 kcal/100 g in the 14% AP sample. Energetic value (kcal) was calculated on the basis of a 100 g portion using Atwater values for fat (9 kcal/g), protein (4 kcal/g), and carbohydrates (4 kcal/g) [38].

The proximate composition of the uncured, reduced-fat chicken sausages formulated with different amounts of fat (25 and 20%) and AP fibre (1 and 2%) was reported also by Choi et al. [18]. The results showed that the addition of apple pomace fibre to the formulation successfully reduced fat content in sausages, while improving quality characteristics relative to control (30% fat). In another paper, Choi et al. [34] showed that the fat content in frankfurters with added makgeolli lees fibre was successfully reduced; in fact, 20% fat frankfurters with the addition of 2% makgeolli lees fibre had quality characteristics similar to those of the control frankfurters (30% fat).

In addition to nutritional composition, we also determined the TPC and antioxidant activity (ABTS, DPPH, and FRAP) of fortified salami (Table 4). All values increased with respect to control, especially when the percentage of AP was 14%; in fact, the TPC increased from 1.12 to 1.74 mg GAE/g, ABTS from 2.19 to 3.10 mg TE/g, DPPH from 1.58 to 2.39 mg TE/g, and FRAP from 0.18 to 0.25 mg TE/g. Generally, it is difficult to make comparisons with data in the literature because the TPC and antioxidant activity are evaluated with the starting AP used for fortification, not with the final fortified products, especially if the functionalised foods are of animal origin. In fact, the few examples are concerned mainly with bakery products or products of plant origin [4]. Jung et al. [39] studied impingement drying for preparing dried AP flour and its fortification in bakery and meat products, and found that wet AP-fortified meat products had significantly higher TPC value and radical scavenging activity, evaluated by DPPH assay, than the control.

### 3.4. Microbiological Analysis of Salami

**Figure 3:**
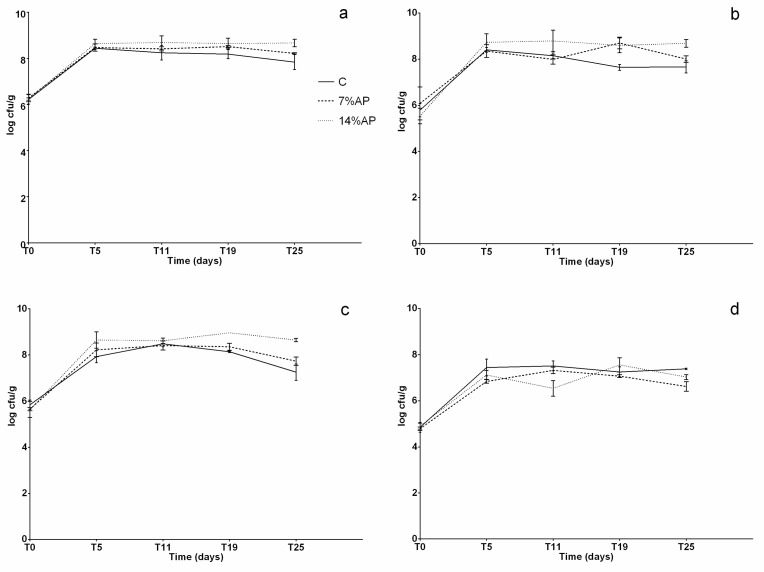
Microbiological analysis of total bacterial count and desirable bacteria during the ripening. (**a**) PCA, plate count agar, total mesophilic aerobic flora; (**b**) MRS, Man, Rogosa and Sharpe agar, *Lactobacillus* spp.; (**c**) M17 agar, *Lactococcus* spp.; (**d**) ENT, enterococcus agar, enterococci.

**Figure 4:**
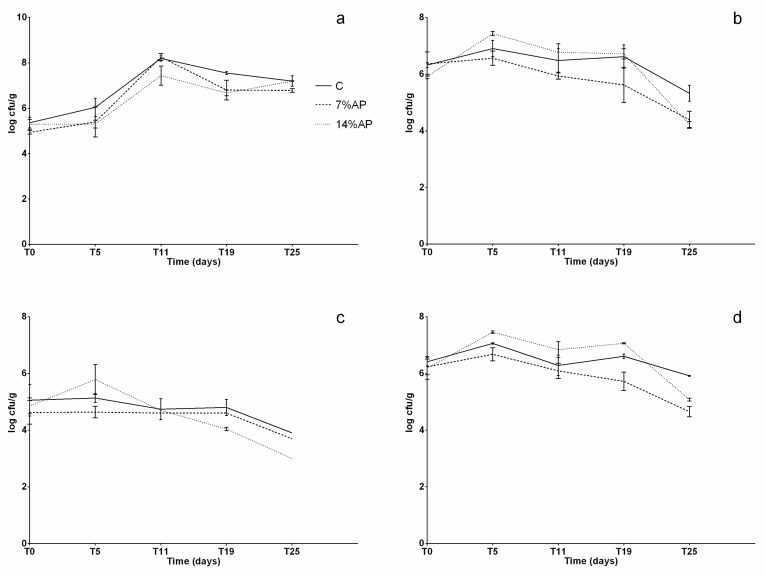
Microbiological analysis of spoilage and potential foodborne pathogen bacteria during the ripening. (**a**) BP, Baird Parker agar, *Staphylococcus* spp.; (**b**) VRBG, violet red bile glucose agar, *Enterobacteriaceae*; (**c**) VRBL, violet red bile lactose agar, total coliforms; (**d**) PS103 pseudomonas agar, *Pseudomonas* spp.

Results of the microbiological analysis are reported in Figure 3a–d and Figure 4a–d.

As for the bacterial populations desirable for a correct ripening of the products, statistically significant differences (*p* < 0.05) were observed for total aerobic mesophilic flora at T25 (C vs. 14% AP); for *Lactobacillus* spp. at T19 (C vs. 7% AP and C vs. 14% AP) and at T25 (C vs. 14% AP); for *Lactococcus* spp. at T5 (C vs. 14% AP), T19 (C vs. 14% AP) and T25 (C vs. 14% AP and 7% vs. 14% AP); for enterococci at T5 (C vs. 7% AP), T11 (C vs. 14% AP and 7% vs. 14% AP), T19 (7% vs. 14% AP) and at T25 (C vs. 7% AP). The bacterial load of these groups at the beginning of the process was about 5 log CFU/g, while at the end of ripening, they reached 10^8–^10^9^ CFU/g. On average, these bacterial populations reached higher values in batches with the addition of apple pomace. This can be linked to the composition of the product, which contained significant amounts of carbohydrate as well as small amounts of proteins, vitamins and minerals [40]. All of these components and simple sugars in particular are good substrates for lactic acid bacteria (LABs) fermentation. As for spoilage bacterial populations, statistically significant differences were observed for *Staphylococcus* spp. at T5 (C vs. 7% and C vs. 14%), at T11 (C vs. 14% and 7% vs. 14%) and at T19 (C vs. 7% and C vs. 14%); for *Enterobacteriaceae* at T5 (7% vs. 14%), at T11 (7% vs. 14%), at T19 (C vs. 7% and 7% vs. 14%) and T25 (C vs. 7% and C vs. 14%); for total coliforms at T5 (7% vs. 14%); for *Pseudomonas* spp. at T5 (7% vs. 14%), at T11 (C vs. 14% and 7% vs. 14%), at T19 (C vs. 7% and 7% vs. 14%) and at T25 (C vs. 7% and C vs. 14%). In general, the bacterial concentration of *Enterobacteriaceae*, total coliforms and *Pseudomonas* spp. at the beginning of the process was about 5 log CFU/g and decreased during the ripening process. No Staphylococcus aureus was detected during the experiment. These results show that replacing a part of meat with apple pomace leads to the production of salami with hygienic and safety characteristics comparable to those produced with the traditional recipe.

Through the isolation and identification of fungal specimens grown on the external surface of salami, two species were identified: *Penicillium cavernicola* and *Candida zeylanoides*. *C. zeylanoides* is a yeast species commonly isolated from the outer surface of aged cheeses (including camembert and blue cheeses). It is a common species in meat product manufacturing and processing plants. *C. zeylanoides*, like many other species belonging to the genus Candida, is an opportunistic pathogen but is only related to nosocomial infections and is not a foodborne pathogen. The genus Penicillium is a strong part of the composite mycobiota that quickly colonize the external part of both industrially produced and handmade salami [41]. It is interesting to note that the growth of *P. cavernicola* was predominant compared to all of the other species on the external part of salami produced with the addition of dry apple pomace.

### 3.5. Sensory Evaluation of Fortified Salami

**Figure 5:**
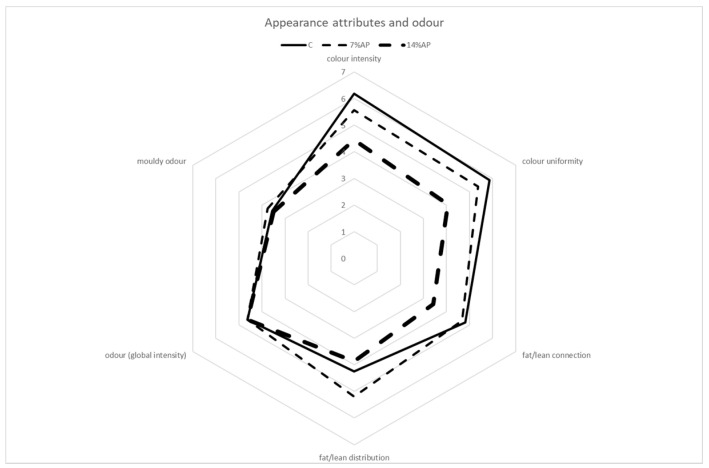
Sensory descriptive analysis. Appearance attributes and odour.

**Figure 6:**
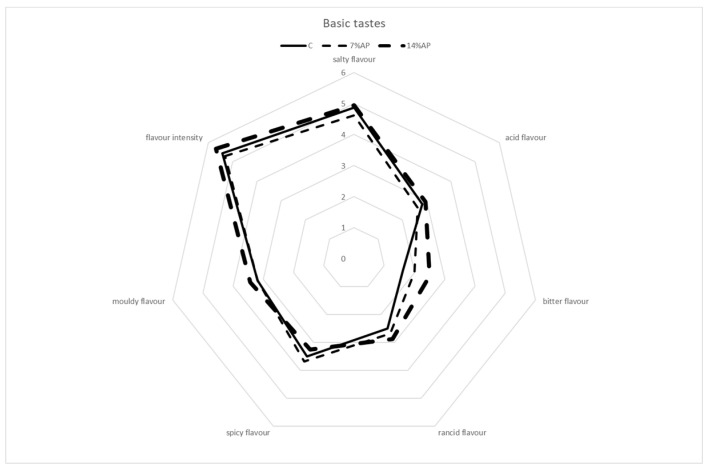
Sensory descriptive analysis. Basic taste.

**Figure 7:**
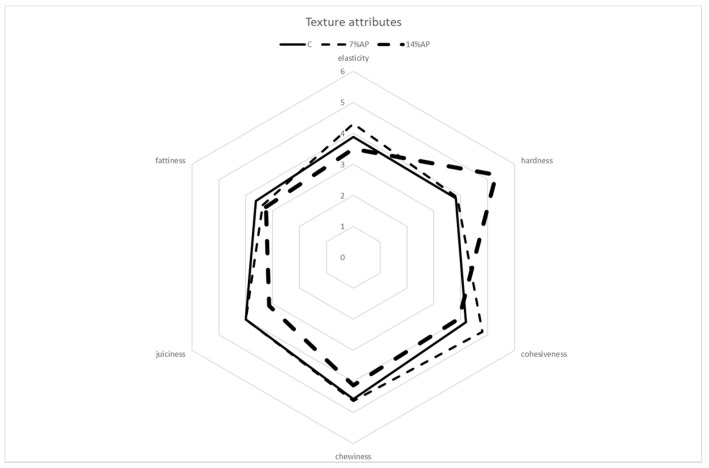
Sensory descriptive analysis. Texture attributes.

The results of the sensory evaluation are shown in Figure 5, Figure 6 and Figure 7.

Among the appearance attributes, higher scores were assigned for the characteristics of colour intensity, colour uniformity and fat/lean distribution in salami produced without the addition of AP (control), followed by those with 7% and finally by those with 14% AP. Regarding fat/lean distribution, the best score was obtained by salami produced with the addition of 7% AP.

The assessors assigned very similar scores to all types of salami for the characteristic odour (global intensity) and mouldy odour. Differences observed in the colour intensity and colour uniformity parameters reflected the results obtained with the colorimetric analysis. As regards basic taste, similar scores were assigned to all samples for the parameters of flavour intensity, salty flavour, acid flavour, mouldy flavour and spicy flavour. Salami produced with the addition of 14% of AP obtained higher scores for bitter flavour and rancid flavour, followed by salami with 7% AP and control salami. Regarding texture attributes, salami produced with the addition of 14% AP obtained lower scores for juiciness and chewiness when compared with salami with 7% AP and control salami. At the same time, it obtained higher scores for hardness. No significant differences were observed for elasticity, cohesiveness and fattiness. Choi et al. (2016) reported that increasing the apple pomace fibre levels from zero to 2% slightly increased hardness, cohesiveness, gumminess, and chewiness [18]. In a review published by Lyu et al. (2020), the authors reported a positive correlation between the addition of apple pomace and the scores for increased texture attributes such as firmness and toughness in various meat products [42]. The highest score for overall acceptability was obtained by the control, followed by salami with 7% AP and then 14% AP. The mean value was 5.88 ± 0.81 for control salami, 5.13 ± 1.15 for salami with 7% AP and 4.63 ± 1.09 for salami with 14% AP. The difference in score between the best and worst batch for overall acceptability was only about 1.25 points; this shows that the replacement of a percentage of meat with apple pomace leads to the production of salamis that are comparable to those obtained with the classic recipe.

## 4. Conclusions

This work confirmed that apple pomace is a valuable by-product with functional properties, useful as a valuable ingredient for the fortification of foods, especially of animal products lacking in dietary fibre and antioxidant phytochemicals such as meat. The enrichment of salami with 7% AP and 14% AP resulted in improved nutritional properties, with lower fat and energy value and higher fibre and antioxidant content, with respect to control. Furthermore, hygiene and safety characteristics were comparable to those of nonfortified products, without leading to alterations and even showing the potential to foster the growth of good bacterial populations such as LABs. The outcome of this study has implications for encouraging the reuse of agri-food waste such as AP and at the same time for successfully improving the nutritional and healthy properties of food, thus increasing the availability on the market of innovative functional products with added value.

## Figures and Tables

**Table 1:** Values for TPC and antioxidant/reducing activity of dried AP (mean values ± SD, n = 3).

	Mean Value ± SD
TPC, mg GAE/g	8.56 ± 0.07
ABTS, mg TE/g	22.13 ± 1.31
DPPH, mg TE/g	11.77 ± 0.33
FRAP, mg TE/g	1.18 ± 0.01

TPC, total phenolic content; GAE, gallic acid equivalents; ABTS, 2,2′-azino-bis(3-ethylbenzothiazoline-6-sulfonic acid) diammonium salt; DPPH, 2,2-diphenyl-1-picrylhydrazyl; FRAP, ferric reducing antioxidant power; TE, Trolox equivalents.

**Table 2:** Phenolic content (mg/kg) in AP (mean values ± SD, n = 3).

Compounds	Mean Value ± SD
quercetin-**3**-*O*-galactoside	1039.54 ± 51.13
quercetin-**3**-*O*-arabinofuranoside	563.69 ± 8.48
phloridzin	396.18 ± 7.92
quercetin-**3**-*O*-rhamnoside (quercitrin)	319.15 ± 6.49
quercetin-**3**-*O*-xyloside	287.72 ± 4.71
quercetin-**3**-*O*-glucoside	42.06 ± 2.38
chlorogenic acid	37.17 ± 2.13
quercetin-**3**-*O*-pentoside	23.13 ± 1.25

**Table 3:** Values of pH, a_w_ and colour coordinates (L*, a* and b*) of fortified salami, sampled at different times (mean values ± SD, n = 3).

	Days	pH	a_w_	Lightness L*	Redness a*	Yellowness b*
7% AP	0	6.31 ± 0.03	0.93 ± 0.06	26.33 ± 2.52	19.67 ± 0.58	18.33 ± 0.58
	5	5.20 ± 0.01	0.93 ± 0.06	26.00 ± 4.00	8.33 ± 0.58	19.67 ± 1.53
	11	5.34 ± 0.02	0.87 ± 0.06	24.00 ± 2.00	9.33 ± 0.58	10.00 ± 0.00
	19	6.62 ± 0.03	0.84 ± 0.06	20.00 ± 1.00	9.00 ± 1.00	12.67 ± 1.53
	25	6.27 ± 0.01	0.77 ± 0.06	23.67 ± 0.58	15.33 ± 0.58	5.33 ± 0.58
14% AP	0	5.95 ± 0.02	0.94 ± 0.00	32.00 ± 2.65	14.33 ± 0.58	19.67 ± 0.58
	5	4.79 ± 0.06	0.93 ± 0.06	33.33 ± 0.58	5.33 ± 0.58	24.33 ± 0.58
	11	4.93 ± 0.06	0.90 ± 0.06	33.33 ± 10.1	11.33 ± 1.16	16.33 ± 2.52
	19	5.55 ± 0.14	0.85 ± 0.06	31.00 ± 0.00	10.67 ± 1.16	13.33 ± 0.58
	25	6.36 ± 0.08	0.75 ± 0.06	19.33 ± 2.08	4.00 ± 0.00	12.67 ± 0.58
C	0	6.48 ± 0.01	0.93 ± 0.06	29.00 ± 2.65	19.00 ± 1.00	18.67 ± 1.16
	5	5.88 ± 0.03	0.93 ± 0.10	26.00 ± 2.00	15.68 ± 0.58	21.33 ± 0.58
	11	6.10 ± 0.03	0.86 ± 0.06	23.00 ± 1.00	14.33 ± 0.58	17.00 ± 0.00
	19	6.29 ± 0.01	0.84 ± 0.10	21.00 ± 6.08	10.33 ± 3.06	10.67 ± 3.51
	25	6.88 ± 0.11	0.79 ± 0.06	20.67 ± 1.16	13.00 ± 0.00	11.67 ± 1.16

7% AP, salami with 7% added AP; 14% AP, salami with 14% added AP; C, control (no added AP, 0%); a_w_, water activity.

**Table 4:** TPC and antioxidant activity (ABTS, DPPH, and FRAP) of fortified salami at the end of ripening (mean values ± SD, n = 3).

	C	7% AP	14% AP
TPC, mg GAE/g	1.12 ± 0.11	1.54 ± 0.12	1.74 ± 0.12
ABTS, mg TE/g	2.19 ± 0.02	2.80 ± 0.17	3.10 ± 0.15
DPPH, mg TE/g	1.58 ± 0.15	2.16 ± 0.04	2.39 ± 0.16
FRAP, mg TE/g	0.18 ± 0.00	0.23 ± 0.00	0.25 ± 0.00

## Data Availability

Data is contained within the article and (Supplementary Material.

## Supplementary Materials

The following supporting information can be downloaded at: https://www.mdpi.com/article/10.3390/antiox11071221/s1, Figure S1: Temperature and relative humidity during fermentation and ripening of the salami. Solid line: temperature (°C), dotted line: relative humidity (%); Figure S2: HPLC-UV chromatographic profile of phenolic compounds in AP extract (1. chlorogenic acid; 2. quercetin-3-*O*-rutinoside (rutin); 3. quercetin-3-*O*-galactoside; 4. quercetin-3-*O*-glucoside; 5. quercetin-3-*O*-xyloside; 6. quercetin-3-*O*-arabinopiranoside; 7. quercetin-3-*O*-arabinofuranoside; 8. quercetin-*O*-pentoside; 9. quercetin-3-*O*-rhamnoside (quercitrin); 10. phloridzin). Figure S3: Instrumental texture profile analysis. C, control; 7%AP, salami with 7% added AP; 14%AP, salami with 14% added AP.
