# Supplementary material for: Apple Pomace as Valuable Food Ingredient for Enhancing Nutritional and Antioxidant Properties of Italian Salami
Source: Antioxidants (Basel). 2022 Jun 22;11(7):1221. doi: 10.3390/antiox11071221 (PMC9311962; doi:10.3390/antiox11071221)
Supplement: Supplementary file 1 [file antioxidants-11-01221-s001.zip › antioxidants-1774194-supplementary.pdf]

SUPPLEMENTARY MATERIAL

Apple Pomace as Valuable Food Ingredient for Enhancing Nutritional and Antioxidant Properties of Italian Salami

[1] Department of Pharmaceutical Sciences, University of Perugia, 06126 Perugia, Italy; luna.pollini@studenti.unipg.it (L.P.); francesca.blasi@unipg.it (F.B,); federica.ianni@unipg.it (F.I.); lina.cossignani@unipg.it (L.C.)
[2] Department of Veterinary Medicine, University of Perugia, 06126 Perugia, Italy; luca.grispoldi@unipg.it (L.G.); beniamino.cencigoga@unipg.it (B.T.C.-G.)
[3] Center for Perinatal and Reproductive Medicine, University of Perugia, Santa Maria della Misericordia University Hospital, 06132 Perugia (Italy); lina.cossignani@unipg.it (L.C.)
* Correspondence: lina.cossignani@unipg.it (L.C.); luca.grispoldi@unipg.it. (L.G.); Tel.: +39-075-585-7959 (L.C.); +39-075-585-7935 (L.G.)

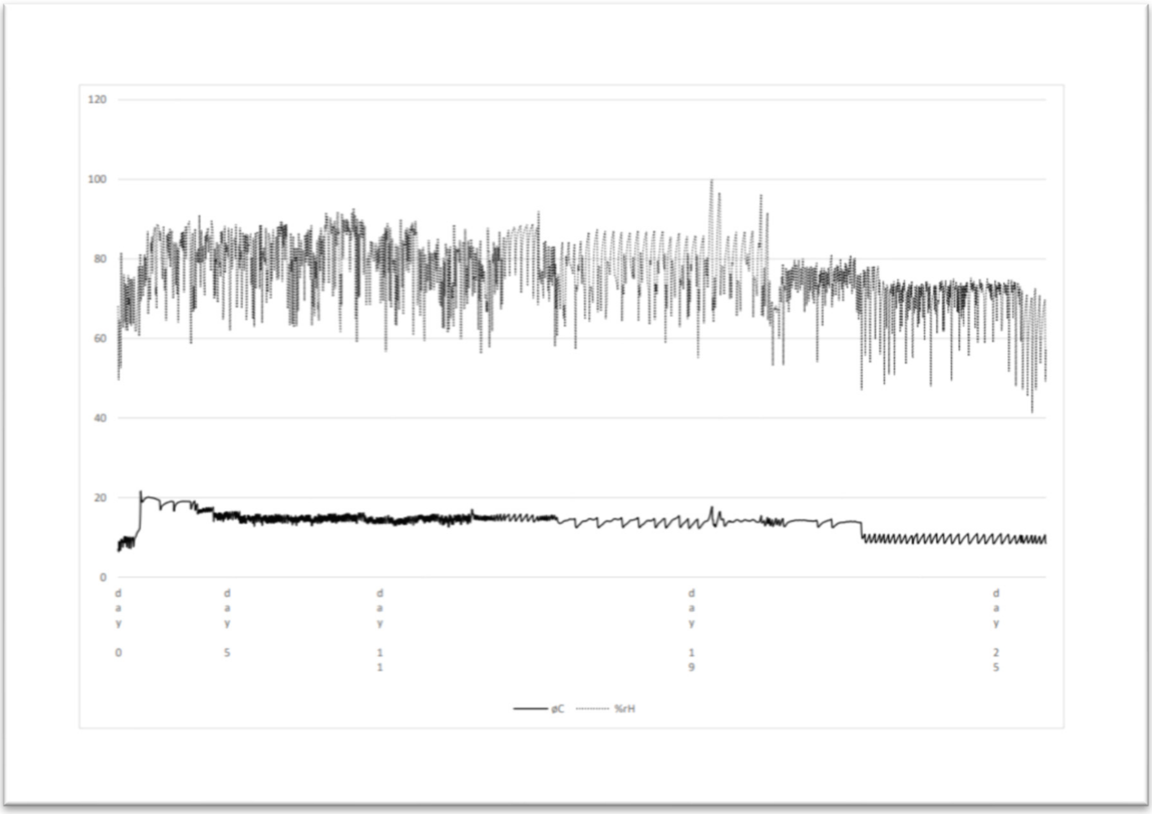

Figure S1: Temperature and relative humidity during fermentation and ripening of the salami. Solid line: temperature (°C), dotted line: relative humidity (%);

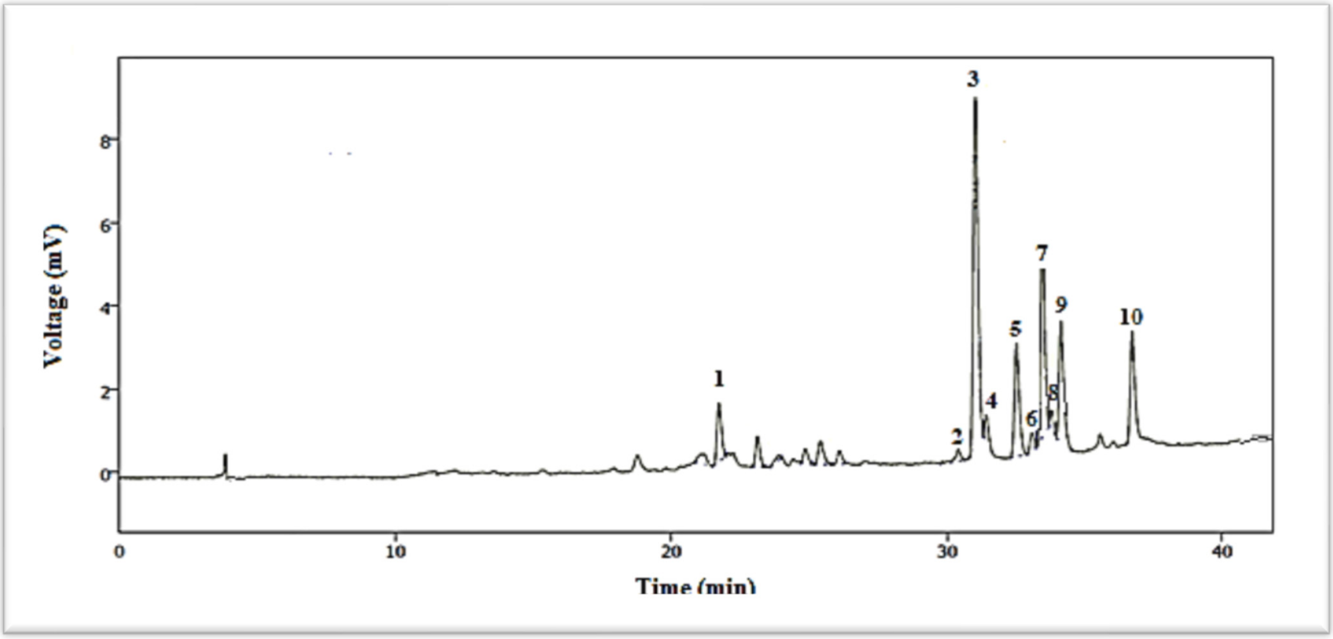

Figure S2: HPLC-UV chromatographic profile of UAE extract.

1 chlorogenic acid

2 quercetin-3-O-rutinoside (rutin)

3 quercetin-3-*O*-galactoside

4 quercetin-3-*O*-glucoside

5 quercetin-3-*O*-xyloside

6 quercetin-3-*O*-arabinopiranoside

7 quercetin-3-*O*-arabinofuranoside

8 quercetin-*O*-pentoside

9 quercetin-3-rhamnoside (quercitrin)

10 phloridzin

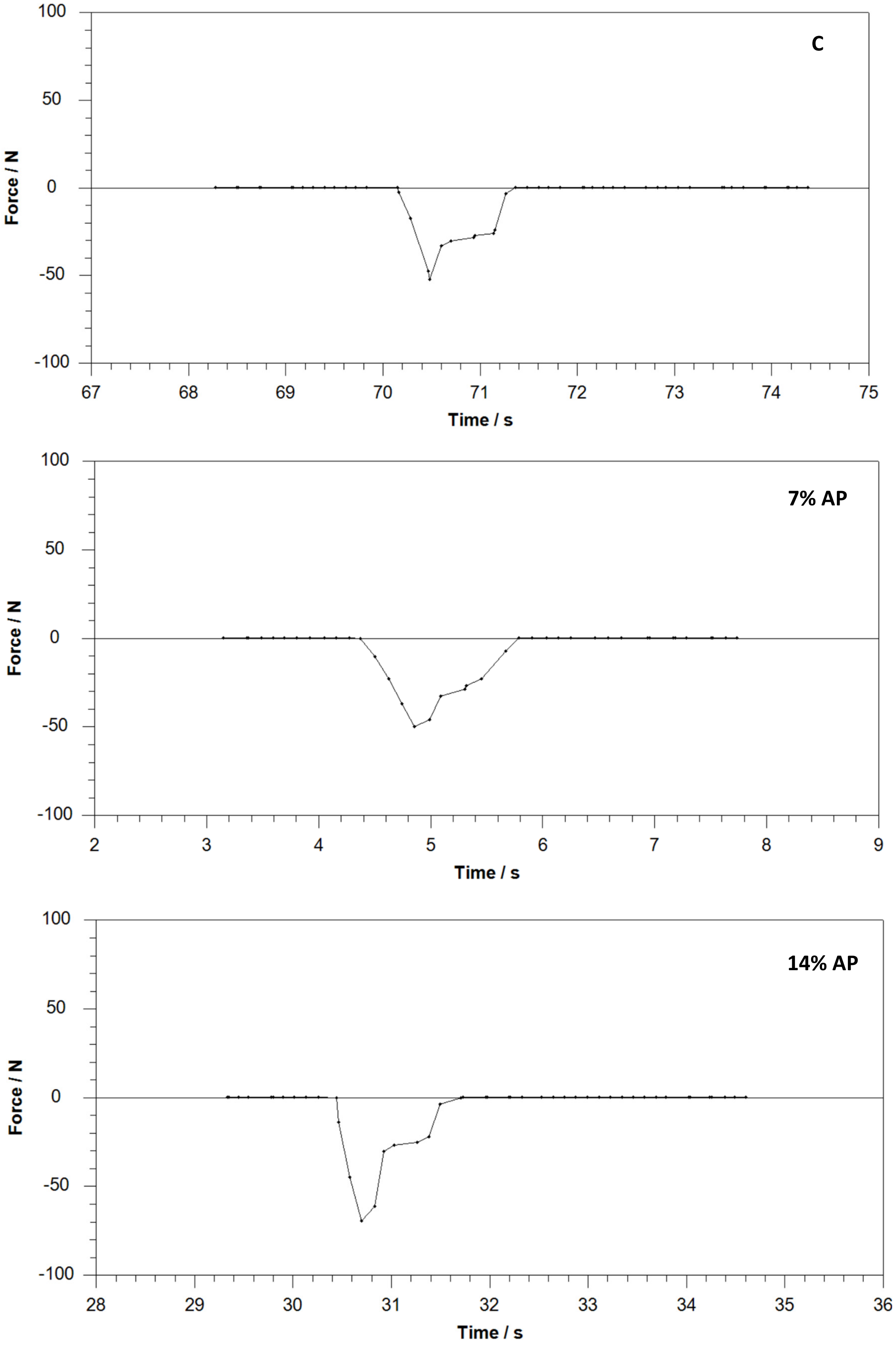

Figure S3: Instrumental texture profile analysis. C, control; 7% AP, salami with 7% added AP; 14% AP, salami with 14% added AP.
